# Severe bleeding events among critically ill patients with haematological malignancies

**DOI:** 10.1186/s13613-024-01383-2

**Published:** 2024-10-07

**Authors:** Clara Vigneron, Clément Devautour, Julien Charpentier, Rudy Birsen, Matthieu Jamme, Frédéric Pène

**Affiliations:** 1 Service de Médecine Intensive-Réanimation, Hôpital Cochin, Assistance Publique - Hôpitaux de Paris, 27 rue du Faubourg Saint Jacques, Paris, 75014 France; 2 Université Paris Cité, Paris, France; 3 Service d’hématologie clinique, Hôpital Cochin, Assistance Publique - Hôpitaux de Paris, 27 rue du faubourg Saint Jacques, Paris, 75014 France; 4 Service de médecine intensive-réanimation, Hôpital Privé de l’Ouest Parisien, Ramsay Générale de Santé, 14 Rue Castiglione del Lago, Trappes, 78190 France; 5 Cardiovascular Epidemiology), Centre de Recherche en Epidémiologie et Santé des Populations, INSERM U-1018, Université de Versailles Saint- Quentin, Team 5 (EpReC, Renal, 16, avenue Paul Vaillant Couturier, Villejuif, 94807 France; 6 Institut Cochin, INSERM U1016, CNRS UMR 8104, Université Paris Cité, 22 rue Méchain, Paris, 75014 France

**Keywords:** Critical care, Haemorrhage, ICU-acquired bleeding, Haematological malignancy, Thrombocytopenia

## Abstract

**Background:**

Bleeding events are common complications in critically ill patients with haematological malignancies. The objective of this study was to assess the incidence and identify determinants of ICU-acquired severe bleeding events in critically ill patients with haematological malignancies. We conducted a single-center retrospective study including all adult patients with a history of haematological malignancy requiring unplanned ICU admission over a 12-year period (2007–2018). The primary endpoint was the occurrence of ICU-acquired (i.e. after the first 24 h in the ICU) severe bleeding events, as defined as grades 3 or 4 of the World Health Organization classification.

**Results:**

A total of 1012 patients were analysed, mainly with a diagnosis of lymphoma (*n* = 434, 42.9%) and leukaemia or myelodysplastic syndrome (*n* = 266, 26.3%). Most patients were recently diagnosed (*n* = 340, 33.6%) and under active cancer treatment within the last 3 months (*n* = 604, 59.7%). The main cause for admission was infection (*n* = 479, 47.3%), but a significant proportion of patients were admitted for a primary haemorrhage (*n* = 99, 10%). ICU-acquired severe bleeding events occurred in 109 (10.8%) patients after 3.0 days [1.0–7.0] in the ICU. The main source of bleeding was the gastrointestinal tract (*n* = 44, 40.3%). Patients experiencing an ICU-acquired severe bleeding event displayed prolonged in-ICU length of stay (9.0 days [1.0–6.0] vs. 3.0 [3.5–15.0] in non-bleeding patients, *p* < 0.001) and worsened outcomes with increased in-ICU and in-hospital mortality rates (55% vs. 18.3% and 65.7% vs. 33.1%, respectively, *p* < 0.001). In multivariate analysis, independent predictors of ICU-acquired severe bleeding events were chronic kidney disease (cause-specific hazard 2.00 [1.19–3.31], *p* = 0.008), a primary bleeding event present at the time of ICU admission (CSH 4.17 [2.71–6.43], *p* < 0.001), non-platelet SOFA score (CSH per point increase 1.06 [1.01–1.11], *p* = 0.02) and prolonged prothrombin time (CSH per 5-percent increase 0.90 [0.85–0.96], *p* = 0.001) on the day prior to the event of interest.

**Conclusions:**

Major bleeding events are common complications in critically ill patients with haematological malignancies and are associated with a worsened prognosis. We identified relevant risk factors of bleeding which may prompt closer monitoring or preventive measures.

## Introduction

Patients with haematological malignancies are prone to life-threatening complications requiring ICU admission, most often related to the underlying disease or its treatment such as cytostatic chemotherapy and immunosuppressive therapy [1]. Depending on the type of malignancy, seven to 22.5% of patients with haematological malignancies may require ICU admission [2, 3], associated with considerable mortality rates ranging from 31 to 47% [1, 4].

Severe bleeding is a common condition in critically ill onco-hematology patients, who exhibit multiple risk factors of bleeding, including coagulation disorders related to bone marrow suppression and peripheral coagulopathy, mucosal damage related to chemotherapy or radiation, malignant organ infiltration, and requirements for invasive procedures [5–11]. Severe haemorrhage thus accounts for a common reason for ICU admission. In a population-based cohort study of 87,965 patients with hematological malignancies in the state of Ontario (Canada), the prevalence of major bleeding at ICU admission was 7.6%, albeit ranging from 5 to 10% across different types of malignancies [12]. Besides, bleeding may occur secondarily as a complication of the ICU stay, though this question has been insufficiently addressed in the literature.

The objective of this study was to assess the incidence and identify determinants of ICU-acquired major bleeding events in critically ill patients with haematological malignancies.

## Methods

### Patients and setting

We conducted a single-center retrospective study in a 24-bed medical ICU in a tertiary care teaching hospital. The study included adult patients (age ≥ 18 years) with a history of hematological malignancy (either diagnosed before or during the ICU stay) requiring unplanned ICU admission from January 2007 to December 2018. Admissions to perform and secure specific procedures were not considered in the absence of acute organ failure. In our hospital, patients undergoing elective surgery are generally hospitalized in a dedicated surgical ICU. Patients in complete remission for more than 5 years were not included. Only the first ICU stay was considered in case of multiple ICU admissions. The study was conducted in accordance with the Helsinki declaration and was approved by the ethics committee of the Société de Réanimation de Langue Française (CE SRLF 24 − 023) which waived the need for signed consent due to its retrospective design.

### Data collection

Clinical and biological data were extracted from the patient data management system (Clinisoft, GE Healthcare) or individually collected from the medical file. The following characteristics of the underlying malignancy were collected: type, stage and status, treatment course, stem cell transplantation and ongoing chemotherapy, radiotherapy, or targeted therapy and immunotherapy within the last 3 months. Severity at admission was assessed by the Simplified Acute Physiology Score 2 (SAPS2) and the Sequential Organ Failure Assessment (SOFA) score calculated within the first 24 h in the ICU [13, 14]. We computed a modified SOFA score without its platelet component (non-platelet (np)-SOFA) at admission and at the time of ICU-acquired bleeding event [15, 16].

During the ICU stay, we collected the daily requirements for organ supports (mechanical ventilation, vasopressors, renal replacement therapy), the severity as assessed by the SOFA and np-SOFA scores, the transfusions of blood products (packed red blood cells, platelet concentrates, fresh frozen plasma) and the time course of biological variables including hemoglobin level, haematocrit, platelet count, leukocyte count, creatinine, urea and bilirubin levels, protidemia, prothrombin time and fibrinogen level. The worst value was retained in case of multiple measurements on one single day.

### Intended management in the ICU

Enteral nutrition was generally initiated on the second day following ICU admission. Proton pump inhibitors were administered in patients unable to receive enteral nutrition and in those with history of gastroduodenal ulcer disease. Depending on the type of invasive procedure, transfusions of blood products were generally indicated by platelet count thresholds of 20 to 50 G/L and prothrombin times of 40–50%. Insertion of central venous catheters was ultrasound-guided with a platelet transfusion threshold of 20 G/L in compressible jugular and femoral sites. The management of bleeding events combined restoration of coagulation functions through transfusion of platelet concentrates, fresh frozen plasma and fibrinogen concentrates as indicated, associated whenever possible with hemostatic procedures through fiberoptic endoscopy, arterial embolization or surgery [17]. Patients were transfused with leukodepleted blood products in accordance with French national and international guidelines [18–20]. Red blood cell transfusions in patients with euvolemic anemia were elicited by a hemoglobin threshold of 7 g/dL [21]. Platelet concentrates were prepared from pooled whole blood donations or from single-donor apheresis, with indications depending on their availability in the blood bank and particular recipient requirements (e.g. anti-HLA immunization). Prophylactic platelet transfusion was triggered by platelet counts of 10 to 20 G/L. Therapeutic platelet transfusion for the treatment of ongoing bleeding was aimed at increasing platelet count above 50 G/L and 100 G/L in case of extracranial and intracranial haemorrhages, respectively. Bleeding events occurring under curative anticoagulant treatment were treated with appropriate antagonization.

### Primary endpoint

The primary endpoint was the occurrence of ICU-acquired (i.e. after the first 24 h in the ICU) recurrent or new-onset severe bleeding events.

### Definitions of severe bleeding events

Severe bleeding events were defined as grades 3 or 4 of the World Health Organization (WHO) classification [22, 23]. Grade 3 accounted for gross blood loss responsible for deterioration of organ failures and/or requiring transfusion of up to two red blood cells concentrates within 24 h of onset. Grade 4 accounted for debilitating blood loss including central nervous system or retinal haemorrhages, massive bleeding requiring transfusion or more than two red blood cells concentrates within 24 h of onset, and fatal bleeding from any source. Severe bleeding events were defined as primary if occurring within the first 24 h from ICU admission, and ICU-acquired when occurring later on. ICU-acquired bleeding events were classified as recurrent from previous primary bleeding or new-onset. The following characteristics of bleeding events were collected: source of bleeding, spontaneous vs. induced by invasive procedures (or anticoagulant overdose), coagulation support, haemostatic management including coagulation support and instrumental procedures (surgery, endoscopic interventions, arterial embolisation).

### Statistical analysis

Continuous variables were expressed as median [interquartile range] and categorical variables as counts (percentages) and compared using the Chi-square method, Fisher’s exact test, the Mann-Whitney U test, and the Student’s t-test when appropriate. Concerning the risk of developing an ICU-acquired major bleeding event, a competitive risk analysis was performed to ascertain associations with occurrence of bleeding event, using cause-specific risk analysis [24]. Concomitant treatments, np-SOFA score and biological covariates were considered as time-dependent covariates. Because np-SOFA and biological covariates could be influenced by the occurrence of ICU-acquired major bleeding event, values obtained the day before analysis were used. Imputation multiple by chained equation was used to handle missing data. Variables with less than 50% of missing data and *p* value < 0.20 in univariate analysis were entered into the multivariate model and checked for possible interactions. Associations were expressed as cause-specific hazard (CSH) with 95% confidence interval. Then, we performed landmark sensitive analysis as recommended for the analysis of time-dependent covariates in time-to-event outcomes [25–27]. We repeated a Cox cause-specific regression at the landmark times day 1, day 2 and day 3 with a 2-day sliding horizon (i.e. day 3, day 4 and day5 respectively). Patients classified as dead or discharged alive did not meet the outcome of ICU-acquired major bleeding event and were considered free of the outcome. At day 2 or day 3, patients who previously met the outcome ICU-acquired major bleeding event were not included in the model. The np-SOFA score and biological covariates obtained the day before the landmark time were used in analysis and averaged if several values (for a landmark set at day 3, admission, day 1 and day 2 values of continuous covariates were averaged).

All statistical tests were two-sided using a type 1 error of 0.05. Analyses were performed using R 3.5.1 statistical software (R foundation for statistical computing Vienna, Austria) and Prism statistical software (GraphPad software by Dotmatics).

## Results

### Characteristics of patients

**Fig. 1:**
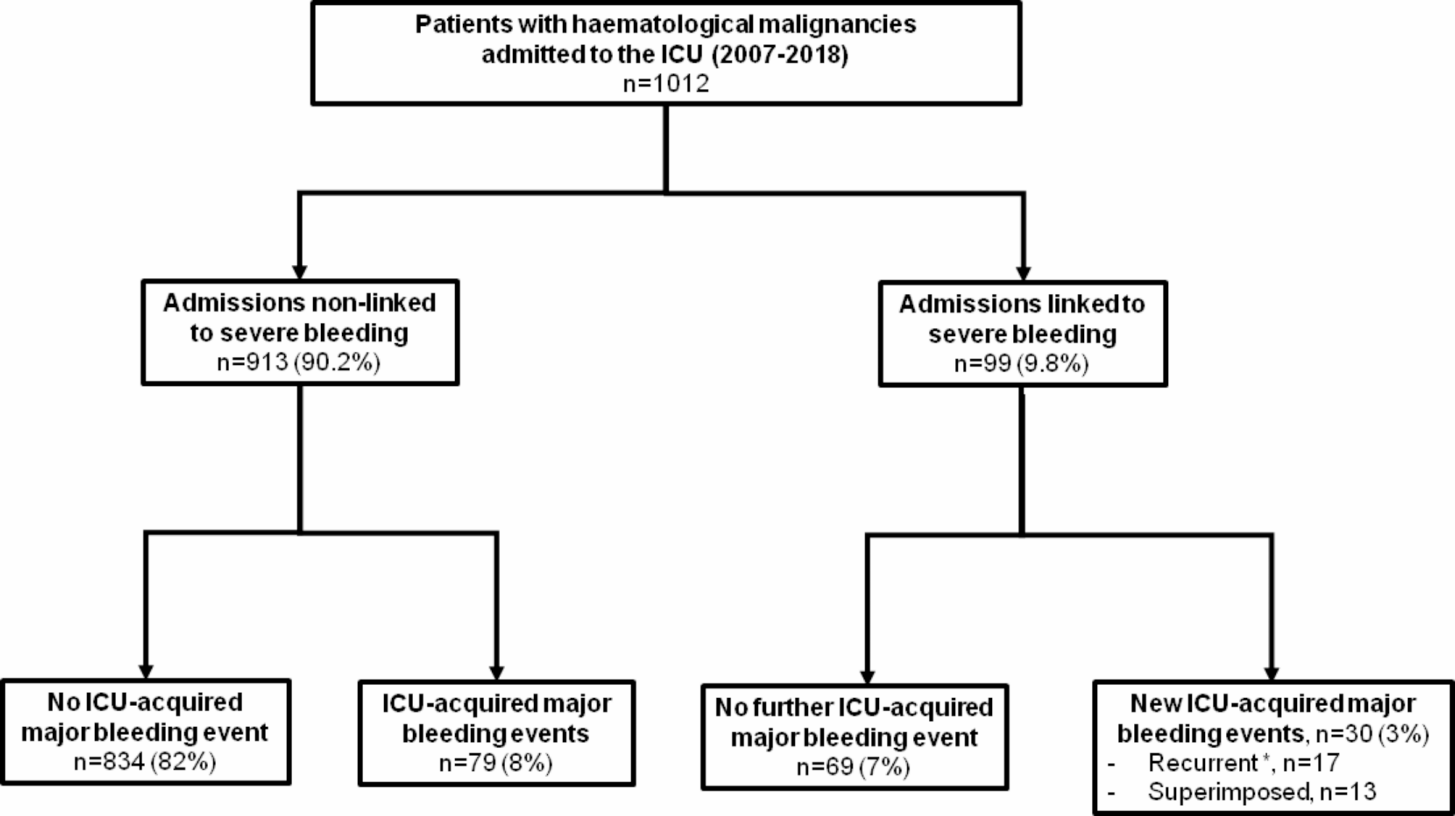
Flow-chart of the study. *Persistent bleeding (*n* = 3), treatment failure (*n* = 8), late recurrence (*n* = 6)

During the 12-year study period, 1012 patients with hematologic malignancies were analysed (Fig. 1). Patients’ characteristics are displayed in the Table 1. Lymphoma, acute leukaemia and myelodysplastic syndrome accounted for the large majority of malignancies. One third of patients were admitted for inaugural complications at the time of the diagnosis of the malignancy. Hence 20% of patients received chemotherapy in the ICU. Ninety nine patients were admitted for the primary reason of bleeding including 40.3% from the gastrointestinal tract, 17% with intracranial haemorrhage, 14% from the lower respiratory tract and 11% with muscular hematoma.

**Table 1:** Characteristics of patients

Characteristics	ICU-acquired severe bleeding event		*p*
	No (*n* = 903)	Yes (*n* = 109)	
**Demographics**			
Age, years	65 [54–76]	65 [57–75]	0.774
Male gender	558 (61.8)	71 (65.1)	0.565
Comorbid conditions and concomitant treatment			
Cirrhosis	21 (2.3)	4 (3.7)	0.598
Chronic Kidney Disease	105 (11.6)	20 (18.3)	0.063
Anticoagulant therapy	191 (21.3)	26 (24.1)	0.589
Antiplatelet therapy	182 (20.3)	19 (17.6)	0.593
**Haematological malignancy**			
Type of malignancy			0.094
Lymphoma	391 (43.3)	43 (39.4)	
Acute leukaemia / myelodysplastic syndrome	227 (25.1)	39 (35.8)	
Myeloma / chronic lymphocytic leukemia	218 (24.1)	19 (17.4)	
Miscellaneous	67 (7.4)	8 (7.3)	
Current haematological status			0.416
Inaugural	297 (32.9)	43 (39.4)	
Partial or complete remission	269 (29.8)	25 (22.9)	
Progression or relapse	231 (25.6)	31 (28.4)	
Chronic phase	105 (11.6)	10 (9.2)	
History of hematopoietic stem cell transplantation	166 (18.4)	29 (26.6)	0.104
Recent hematological treatment (< 3 months)	540 (59.8)	64 (58.7)	0.909
**Features at ICU admission**			
Main cause of admission			
Infection	435 (48.2)	44 (40.4)	0.150
Major bleeding	69 (7.6)	30 (27.5)	< 0.001
Acute kidney Injury	339 (37.5)	62 (56.9)	< 0.001
SOFA score, points	5.0 [3.0–8.0]	8.0 [5.0–11.0]	< 0.001
np-SOFA score, points	3.0 [2.0–6.0]	6.0 [4.0–9.0]	< 0.001
SAPS 2, points	45.0 [35.0–58.0]	57.0 [45.0-77.75]	< 0.001
Platelet count, G/L	79.0 [32.0-180.0]	54.0 [22.0-128.0]	0.004
Hematocrit level, %	27.05 [24.00–33.00]	26.00 [23.00–30.00]	0.036
Creatinine, μmol/L	102.0 [68.0-163.0]	129.0 [80.0-216.0]	0.005
Urea, mmol/L	8.7 [5.7–14.0]	10.6 [6.5–19.7]	0.008
Bilirubin, μmol/L	12.00 [7.00–20.00]	17.00 [8.00-41.25]	< 0.001
Prothrombin time, %	72.0 [58.0-84.5]	62.0 [49.0–75.0]	< 0.001
aPTT ratio	1.17 [1.03–1.34]	1.25 [1.06–1.54]	0.002
Plasma protein level, g/L	60.0 [52.0–67.0]	56.0 [49.75–65.25]	0.016
**Characteristics of the ICU stay**			
Invasive mechanical ventilation	271 (67.8)	83 (90.2)	< 0.001
Vasopressor therapy	290 (32.2)	74 (67.9)	< 0.001
Renal replacement therapy	190 (21.0)	51 (46.8)	< 0.001
In-ICU chemotherapy	180 (19.9)	31 (28.4)	0.052
ICU length of stay, days	3.0 [1.0–6.0]	9.0 [3.5–15.0]	< 0.001
**Outcomes**			
In-ICU mortality	165 (18.3)	60 (55.0)	< 0.001
In-hospital mortality *	277/836 (33.1)	72/108 (65.7)	< 0.001
One-year mortality *	500 (55.6)	86/107 (80.4)	< 0.001
Decisions to forgo life-sustaining therapies	140 (16.6)	37 (33.9)	< 0.001

Variables are expressed as median (interquartile range) or number (percentage) as appropriate. Abbreviations: aPTT: activated partial thromboplastin time; ICU: intensive care unit; np-SOFA: non-platelet Sepsis-related Organ Failure Assessment; SAPS 2: Simplified Acute

Physiology Score 2. * Vital status at hospital discharge and at one year were unavailable for 10 (0.1%) and 8 (0.1%) patients, respectively

### Recurrent and superimposed ICU-acquired major bleeding events

ICU-acquired severe bleeding events occurred in 109 (10.8%) patients after 3.0 days [1.0–7.0] in the ICU, and were associated with an increase of 1 [0–3] point in the np-SOFA score and the requirement for the transfusion of 2 [2-4] packed red cells (Table 2). Among 99 patients admitted with primary haemorrhage, 17 exhibited bleeding recurrence from the same source, whereas 13 exhibited new-onset bleeding from an alternative source. The main source of ICU-acquired bleeding was the gastrointestinal tract (*n* = 44, 40%), with a relatively delayed occurrence (6.0 [2-13] days from ICU admission). The majority of patients with gastrointestinal bleeding (58%) were not receiving enteral nutrition at the time of event. We did not identify any interaction with the prior use of proton pomp inhibitor (56.3% vs. 56.8% in patients with and without gastrointestinal bleeding, *p* = 0.95). Upper digestive endoscopy was performed in 37 patients and made the definite diagnosis of upper gastrointestinal bleeding in 27 patients. Bleeding from lower respiratory tract (*n* = 14, 13%) was mainly diagnosed in invasively ventilated patients, and was associated with significant deterioration in their respiratory conditions, as manifested by an increase of 1 [1] point in the respiratory SOFA component and the requirement for new intubation in six patients. A number of patients were under concurrent curative anticoagulant treatment (23.9% including 6.4% with overdose) or antiplatelet agents (13.8%). Biological records on the day of bleeding showed impaired coagulation parameters including thrombocytopenia (platelet count 41.0 G/L [17.0–90.0]) and prolonged prothrombin time (61.5% [46.8–73.3]). The bleeding event was considered as the primary cause of death in 24 (22%) patients. A further recurrence of bleeding was observed in 34 (31.2%) patients. Patients experiencing ICU-acquired severe bleeding event had prolonged in-ICU length of stay (9.0 days [1.0–6.0] vs. 3.0 [3.5–15.0] in non-bleeding patients, *p* < 0.001). Patients with ICU-acquired bleeding displayed worsened outcomes with dramatic increases in in-ICU and in-hospital mortality rates (55% vs. 18.3% and 65.7% vs. 33.1%, respectively, *p* < 0.001). Patients with intracranial haemorrhage exhibited the highest mortality rate of 77%.

**Table 2:** Characteristics of ICU-acquired major bleeding events

Characteristics	*n* = 109
Time from ICU admission, days	3.0 [1.0–7.0]
WHO bleeding grade	
Grade 3	46 (42.2)
Grade 4	63 (57.8)
Main sources of bleeding	
Gastrointestinal tract	44 (40.3)
Lower respiratory tract	14 (13)
Intravascular catheter	13 (11.9)
Intracranial	12 (11.0)
Miscellaneous	26 (24)
Bleeding related to invasive procedure	24 (22)
Concomitant treatment	
Antiplatelet agent	15/108 (13.8)
Curative anticoagulant	26/108 (23.9)
Corticosteroid	49 (45.0)
Proton pump inhibitor	61 (56.0)
Biological parameters on the day of bleeding event	
Haemoglobin, g/dL	8.1 [7.2–9.3]
Haematocrit, %	26 [23–30]
Platelet count, G/L	41.0 [17.0–90.0]
Creatininemia, μmol/L	108.0 [67.0-201.0]
Uremia, mmol/L	11.9 [7.0-20.6]
Bilirubinemia, μmol/L	26.0 [11.5–87.0]
Prothrombin time, %	61.5 [46.8–73.3]
aPTT ratio	1.31 [1.09–1.71]
Fibrinogen, g/L	3.5 [2.3–5.5]
Serum protein level, g/L	53.0 [45.0–58.0]
Organ support on the day of bleeding event	
Vasopressor therapy	44 (40.4)
Invasive mechanical ventilation	58 (53.2)
Renal replacement therapy	36 (33.0)
np-SOFA score, points	7 (5–12)
np-SOFA score variation from the eve, points	+ 1 (0–3)
Bleeding event management	
Instrumental intervention	35 (32.1)
Surgery	12 (11.0)
Red blood cell transfusion	97 (89.0)
number of units	2.0 [2.0–4.0]
Platelet transfusion	71 (66.1)
number of units	1.0 [0.0–2.0]
Plasma transfusion	36 (33.0)
number of units	0.0 [0.0–2.0]
**Outcomes**	
Further recurrence of bleeding	34 (31.2)
In-ICU mortality	60 (55.0)
Bleeding-attributable mortality	24 (22)
In-hospital mortality	72/108 (65.7)

Variables are expressed as median (interquartile range) or number (percentage) as appropriate. Abbreviations: aPTT: activated partial thromboplastin time; ICU: intensive care unit, np-SOFA: non-platelet Sepsis-related Organ failure Assessment

### Determinants of secondarily acquired major bleeding event

We compared the features of patients with and without ICU-acquired bleeding events (Table 1). Patients with acquired bleeding were more often admitted for a primary bleeding event (27.5% vs. 8.7%, *p* < 0.001), exhibited higher severity scores at ICU admission and therefore required increased organ supports. At ICU admission, patients with further ICU-acquired bleeding exhibited altered coagulation parameters, including decreased platelet count, prolonged prothrombin time and activated partial thromboplastin time (aPTT), as well as lower haematocrit and higher urea level both likely to impact on primary haemostasis.

In multivariate analysis, independent predictors of ICU-acquired severe bleeding event were chronic kidney disease (CSH 2.00 [1.19–3.31], *p* = 0.008) and a primary bleeding event present at the time of ICU admission (CSH 4.17 [2.71–6.43], *p* < 0.001), np-SOFA score (CSH per one-point increase 1.06 [1.01–1.11], *p* = 0.02) and prolonged prothrombin time (CSH per 5-percent increase 0.90 [0.85–0.96], *p* = 0.001) on the day prior to the event of interest (Table 3). Of note, platelet count was not an independent predictor of bleeding.

**Table 3:** Factors associated with acquired major bleeding event in ICU

Characteristics	Univariate analysis CSH [95% CI]	*p*	Multivariate analysis CSH [95% CI]	*p*
**Demographic, hematologic and ICU admission characteristics**				
Age (+ 1 year)	1.04 [0.92–1.18]	0.55	NA	NA
Male gender	1.09 [0.73–1.62]	0.67	NA	NA
Comorbid condition or concurrent treatment				
Chronic Kidney Disease	1.84 [1.13–2.99]	0.01	2.00 [1.19–3.31]	0.008
Antiplatelet therapy	0.79 [0.48–1.30]	0.36	NA	NA
Anticoagulant therapy	1.18 [0.76–1.84]	0.47	NA	NA
Main cause of admission				
Primary bleeding event	4.07 [2.66–6.22]	< 0.001	4.17 [2.71–6.43]	< 0.001
**ICU characteristics (on the day prior to event)**				
np-SOFA (per point)	1.08 [1.04–1.13]	< 0.001	1.06 [1.01–1.11]	0.02
Platelet count (+ 10 G/L)	0.97 [0.94–0.99]	0.01	0.98 [0.96–1.01]	0.20
Haematocrit (+ 1%)	0.94 [0.90–0.98]	< 0.001	0.96 [0.92–1.01]	0.10
Urea (+ 10 mmol/L)	1.29 [1.08–1.55]	0.005	1.14 [0.92–1.43]	0.21
Bilirubin (+ 10 μmol/L)	1.03 [1.01–1.05]	0.01	NA	NA
Prothrombin time (+ 5%)	0.87 [0.82–0.92]	< 0.001	0.90 [0.85–0.96]	0.001

Abbreviations: CSH: cause-specific hazard; ICU: intensive care unit; NA: not analyzed; np-SOFA: non-platelet Sepsis-related Organ Failure Assessment. As a component of the np-SOFA, bilirubin was not entered into the multivariate model

**Fig. 2:**
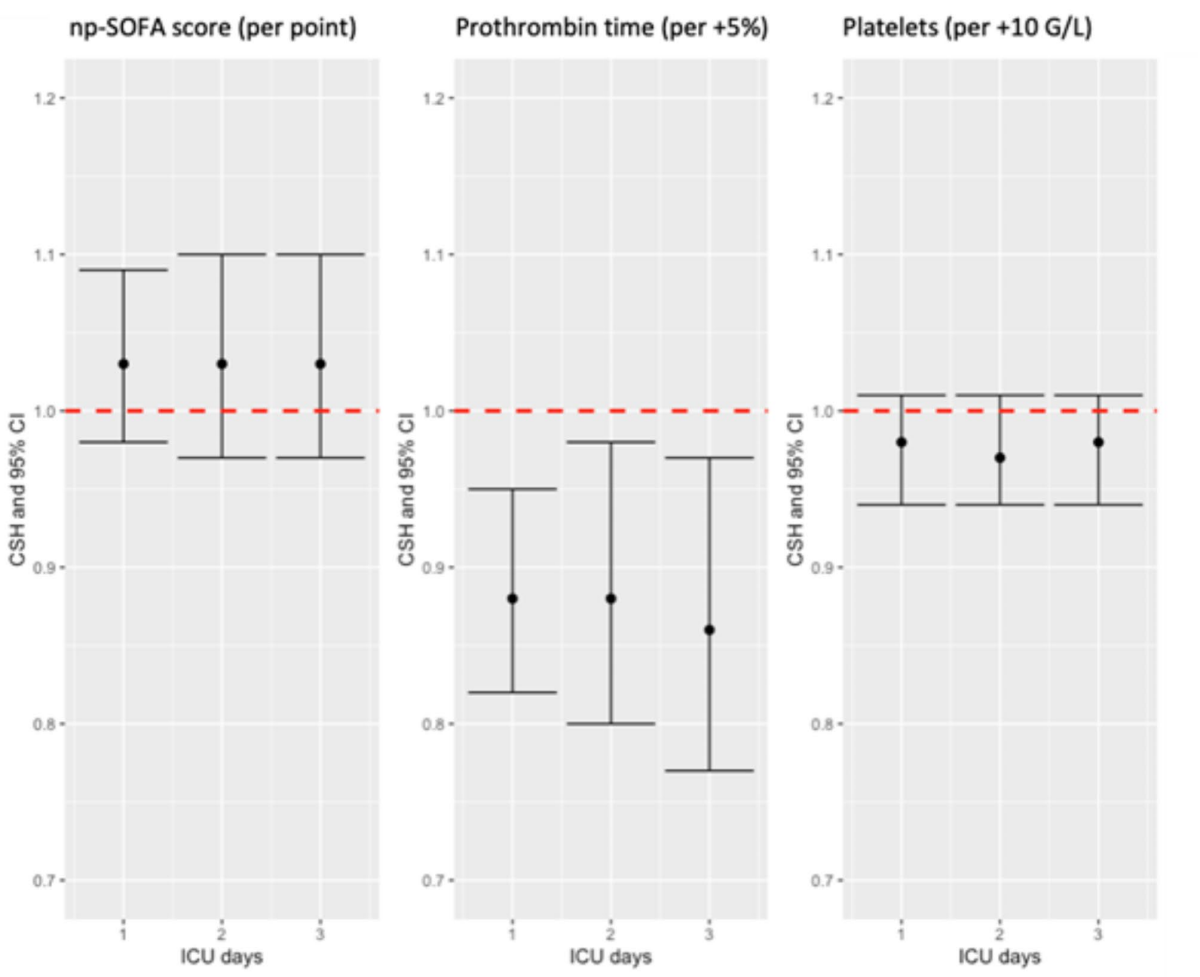
Landmark-sensitive analysis of covariates associated with further bleeding in ICU. The figure represents the cause-specific hazards and their 95% confidence intervals of ICU-acquired severe bleeding events related to np-SOFA score, prothrombin time and platelet count in patients alive at days 1, 2 and 3 after ICU admission, in multivariate analysis adjusted on chronic kidney disease, primary major bleeding at admission, haematocrit and urea. Abbreviations: 95%CI: 95% confidence interval. CSH: ICU: intensive care unit. np-SOFA: non-platelet Sepsis-related Organ Failure Assessment

ICU-acquired severe bleeding is a time-dependent event. In order to take into account the immortal time bias, we addressed the risk factors for further bleeding (mean values since ICU admission) through competitive risk analysis at various landmarks (days 1, 2 and 3 from ICU admission) in patients remaining alive in the ICU. In landmark analysis, the respective CSH related to np-SOFA, platelet count and prothrombin time with regard to further bleeding events are displayed in the Fig. 2. Only prothrombin time was independently associated with severe bleeding event at the three analysed landmarks.

## Discussion

The prognosis of critically ill patients is often appreciated from the primary reason for ICU admission, but it is noteworthy that it is often driven by further ICU-acquired complications. This study provides a comprehensive assessment of bleeding events in a general cohort of ICU hematological patients. ICU-acquired severe bleeding events accounted for a frequent complication affecting about 10% of patients in this setting and were associated with high mortality, often as the primary cause of death. The determinants of bleeding herein identified (chronic kidney disease, prior recent bleeding event, the extent of organ failures and prolonged prothrombin time) characterize a subgroup at risk.

Thrombocytopenia is a hallmark biological disorder in critically ill patients with hematological malignancies through multiple hypoproliferative and peripheral consumption mechanisms, and previous studies indeed have focused on thrombocytopenic patients owing to a presumably higher risk of bleeding [15, 28]. In a cohort of critically ill patients with acute leukaemia and myelodysplastic syndrome, hence with prolonged and profound thrombocytopenia, Russell and al. reported that one third of them experienced a severe or debilitating (WHO grade 3 or 4) bleeding, either at the time of ICU admission for half of them, or secondarily acquired for the others [9]. Interestingly, the risk of bleeding was linked to the depth of thrombocytopenia on admission. The prospective multicentre PLOT-ICU study reported an association between profound thrombocytopenia and severe bleeding in general population of adult ICU patients [29]. In the present study, thrombocytopenia assessed as a dynamic variable over time was not retained as an independent risk factor of bleeding. It is possible that substitutive platelet transfusion was able to mitigate the risk of bleeding in thrombocytopenic patients, even though post-transfusion platelet increments are frequently poor [15, 30]. Nonetheless, platelet transfusion refractoriness was associated with increased risk of ICU-acquired bleeding in patients with hypoproliferative thrombocytopenia [15].

In contrast, prolonged prothrombin time was the only coagulation parameter associated with severe bleeding, in line with previous studies [28, 31, 32]. Prolonged prothrombin time may reflect active coagulopathy and/or decreased production of coagulation factors because of liver failure or interactions of broad-spectrum antibiotics with vitamin K biosynthesis. Prolonged prothrombin time at ICU admission was already identified as an independent risk factor of mortality in onco-hematology patients with thrombocytopenia, but the relation with subsequent bleeding events was not investigated [33]. In a cohort of patients with acute myeloid leukaemia and myelodysplastic syndrome, an increase in INR value was an independent predictor of 24-hour and 5-day bleeding in the ICU, after adjustment on other coagulation parameters with platelet count [28].

We also identified chronic renal failure as an independent predictor of ICU-acquired bleeding, suggesting that uremia may contribute to coagulation alterations, even though the urea level measured right before the event was finally not retained in the multivariate analysis combined with other related covariates. Increased urea levels may also result from upper gastro-intestinal bleeding. In a retrospective review on 2,942 thrombocytopenic onco-hematology patients, the risk of serious haemorrhage appeared not correlated with platelet count, but was independently associated with uremia > 8.2 mmol/L. Of note, the most potent predictor of bleeding in this study was a recent grade 2-3-4 haemorrhage within the five preceding days [34].

The identification of a high-risk population may allow targeted diagnostic and therapeutic interventions. With regard to complex hemostasis alterations, rotational viscoelastic assays involve all components of the coagulation cascade and may provide a functional assessment of clot formation, stabilization and lysis. Although conveniently available as point-of care, such assays did not add to the prediction of bleeding or thrombosis in hematology patients when compared to routine coagulation parameters [28].

This study had several limitations. This was a single-center study based on a retrospective design potentially associated with measurement bias and missing data. So the study can only report associations but not causation. Although modifications in practices may have occurred during this 12-year study, neither the management of hemorrhagic events nor the indications for transfusions did fundamentally change over the past decade, and we did not identify any association with the year of ICU admission. With respect to upper gastrointestinal haemorrhage, we could not carry out a comprehensive analysis to investigate the protective roles of enteral nutrition and treatment with proton pump inhibitors [35]. It should be mentioned that our current management of acute renal failure has recently evolved towards more delayed initiation of renal replacement therapy, with higher serum urea thresholds likely to impact on coagulation [36]. However trials about the timing of initiation of renal replacement therapy reported similar haemorrhage incidences regardless of the allocation arm. Despite the decent size of the cohort, the number of events of interest was relatively low and limited the number of variables entered into the multivariate statistical analysis.

## Conclusion

Major bleeding events are common complications in critically ill patients with haematological malignancies and are associated with a worsened prognosis. A prior recent episode of bleeding should raise caution, since predictive of recurrent or new-onset bleeding. Whether patients are amenable to preventive strategies through transfusion of platelets or fresh frozen plasma should be addressed in a prospective manner. We identified relevant risk factors of bleeding: although some of them are inherent characteristics of patients, our study also revealed relevant biological and clinical features that could help clinicians to better identify patients at high risk of major bleeding event to initiate closer monitoring or preventive measures.

## Data Availability

The datasets generated and/or analysed during the current study are not publicly available due to French regulations but are available from the corresponding author on reasonable request.

## Abbreviations

aPTT: Activated Partial Thromboplastin Time
CSH: Cause-Specific Hazard
ICU: Intensive Care Unit
INR: International Normalized Ratio
np-SOFA: Non-platelet Sequential Organ Failure Assessment
SAPS2: Simplified Acute Physiology Score 2
SOFA: Sequential Organ Failure Assessment
WHO: World Health Organization
